# Plasticity of Migrating CD1b^+^ and CD1b^-^ Lymph Dendritic Cells in the Promotion of Th1, Th2 and Th17 in Response to *Salmonella* and Helminth Secretions

**DOI:** 10.1371/journal.pone.0079537

**Published:** 2013-11-05

**Authors:** Michel Olivier, Benjamin Foret, Yves Le Vern, Dominique Kerboeuf, Laurence A. Guilloteau

**Affiliations:** 1 UR1282 Infectiologie et Santé Publique, INRA, Nouzilly, France; 2 Laboratoire de Cytométrie, Institut National de la Recherche Agronomique, Nouzilly, France; International Center for Genetic Engineering and Biotechnology, India

## Abstract

Dendritic cells (DCs) are pivotal in the development of specific T-cell responses to control pathogens, as they govern both the initiation and the polarization of adaptive immunity. To investigate the capacities of migrating DCs to respond to pathogens, we used physiologically generated lymph DCs (L-DCs). The flexible polarization of L-DCs was analysed in response to *Salmonella* or helminth secretions known to induce different T cell responses. Mature conventional CD1b^+^ L-DCs showed a predisposition to promote pro-inflammatory (IL-6), pro-Th1 (IL-12p40) and anti-inflammatory (IL-10) responses which were amplified by *Salmonella*, and limited to only IL-6 induction by helminth secretions. The other major population of L-DCs did not express the CD1b molecule and displayed phenotypic features of immaturity compared to CD1b^+^ L-DCs. *Salmonella* infection reduced the constitutive expression of TNF-α and IL-4 mRNA in CD1b^-^ L-DCs, whereas this expression was not affected by helminth secretions. The cytokine response of T cells promoted by L-DCs was analysed in T cell subsets after co-culture with *Salmonella* or helminth secretion-driven CD1b^+^ or CD1b^-^ L-DCs. T cells preferentially expressed the IL-17 gene, and to a lesser extent the IFN-γ and IL-10 genes, in response to *Salmonella*-driven CD1b^+^ L-DCs, whereas a preferential IL-10, IFN-γ and IL-17 gene expression was observed in response to *Salmonella*-driven CD1b^-^ L-DCs. In contrast, a predominant IL-4 and IL-13 gene expression by CD4^+^ and CD8^+^ T cells was observed after stimulation of CD1b^+^ and CD1b^-^ L-DCs with helminth secretions. These results show that mature conventional CD1b^+^ L-DCs maintain a flexible capacity to respond differently to pathogens, that the predisposition of CD1b^-^ L-DCs to promote a Th2 response can be oriented towards other Th responses, and finally that the modulation of migrating L-DCs responses is controlled more by the pathogen encountered than the L-DC subsets.

## Introduction

Dendritic cells (DCs) are pivotal in the development of specific T-cell responses to control the diverse types of micro-organisms that invade hosts, as they govern both the initiation and the polarization of adaptive immunity. DCs promote the development of functionally different effector T helper cells including Th1, Th2, Th17 and T regulatory cells. The current paradigm of DC-driven activation of naive Th cells is based on the requirement of three signals provided by pathogens interacting with DCs. The first results from pathogen-derived peptides, presented by MHC II on the DC cell surface, ligating to T-cell receptors, the second requires T-cell co-stimulation molecules, and the third depends on the Th polarizing capacities of DC-derived molecules [1]. The expression of these molecules is dependent on and imprinted by the binding of pathogens to selective pattern recognition receptors (PRR) on immature DCs resulting in their programming during maturation. The role of the different DCs subsets in presenting antigens to T cells is a complex process. Migratory and lymphoid-organ-resident DC subsets with distinct functional abilities have been described [2,3]. 

The plasticity of mouse and human DC subset responses have been demonstrated, at least *in vitro*, through the priming signals from pathogens. Basically, DCs exposed to intracellular pathogens promote a Th1 response. In *Salmonella* infection, specific Th1 cells that produce IFN-γ are essential for controlling bacterial growth [4,5]. Both Th1 and B cells contribute to protecting against *Salmonella* [5,6]. More recently, it has been shown that *Salmonella enterica* serovar Enteritidis infection is able to induce antigen-specific IL17A production by CD4^+^ Th17 cells and γδ^+^ T cells and other CD4^-^ lymphocytes [7]. In contrast, helminth infestation and free-living helminths are known to drive a dominant Th2 response associated mainly with IL-4 and IL-13 production by CD4^+^ T cells [8]. The plasticity of DC subsets could also be tuned by the microenvironment which includes tissue-derived factors as cytokines and determines the T helper commitment. 

To investigate the plasticity of migrating lymph DCs (L-DCs), we used physiologically generated DCs collected from a pseudo-afferent lymphatic cannulation model [9,10]. CD1b^+^ L-DCs have been described, in a previous study, with phenotypic features of conventional mature DCs, including functional abilities to uptake soluble antigens and *Salmonella*. They have been shown to prime naive T cells associated with IFN-γ and IL-10 production. The T-cell subset activated by *Salmonella* clearly comprised the CD4^+^ T cells, and CD1b^+^ L-DCs directed the cytokine responses towards both IFN-γ and IL-10. Moreover, CD1b^+^ L-DCs were potentially able to induce IFN-γ and IL-4 responses in an allogeneic reaction [11]. This suggests the potential of these migrating physiologically generated mature L-DCs to modulate their response according to the antigen encountered.

To investigate this hypothesis, we characterized the CD1b^+^ L-DCs response to *Salmonella* and to helminth secretions, and consequently their potential to promote the cytokine response by T cells. We also characterized the other major population of migrating lymph antigen-presenting cells which does not express the CD1b molecule and assessed their ability to respond to *Salmonella* and helminth secretions. 

## Materials and Methods

### Ethics statement

The animal experiments were conducted under a license issued by the Direction des Services Vétérinaires of Tours (accreditation B-37-175-3) and were approved by the Regional Centre-Limousin Ethics Committee (CL2006-012). 

### Surgery and lymph collection

Prealpes du sud ewes (one to four years old) originating from the Unité Commune d’Expérimentation Animale (INRA, Jouy-en-Josas, France) or from the Plateforme d’Infectiologie Expérimentale (PFIE) (INRA, Nouzilly, France), were housed in the PFIE for surgery and lymph collection. They were born and raised in salmonellosis and helminth-free herds. Prescapular and cervical lymph duct cannulation were performed as described previously [11]. Lymph was collected continuously and cells were frozen and stored in liquid nitrogen. Over 95% viability was obtained after thawing.

### Lymph DC cells (L-DCs) phenotyping and sorting

Lymph cells were thawed, washed and adjusted to 5 x 10^6^ cells/ml of RPMI 1640 medium (Life Technologies, Cergy Pontoise, France) supplemented with 10% fetal calf serum (FCS), 2mM L-glutamine, 1mM sodium pyruvate, and 50 μM ß-mercaptoethanol, 20 IU/ml of penicillin and 20 μg/ml of streptomycin (complete medium). 

The CD1b^+^ L-DCs subset was characterized elsewhere [11], the phenotype of the CD1b^-^ L-DCs subset was defined in this study by labelling cells with the same set of monoclonal antibodies (mAbs) used under the same conditions as described previously [11]. Cells were first incubated with a mixture of mAbs including anti-ruminant CD4 (17D1), CD8, γδ TCR, CD45R and CD1b (2µg/ml of each mAb for 1 x 10^8^ cells) for 20 min followed by R-Phycoerythrin (RPE)-conjugated F(ab’)_2_ fragment goat anti-mouse (GAM) IgG (1:200) (Jackson ImmunoResearch, Suffolk, UK). Cells were then incubated with one of the primary or appropriate isotype control mAbs followed by either Alexa Fluor® 488-conjugated GAM IgG1 (1:600) (Molecular Probes, Eugene, OR) or FITC-conjugated GAM IgG2a (1:200) (Caltag Laboratories, Burlingame, CA). After washes, cells were resuspended and fixed in 100 µl of 1% paraformaldehyde in buffered saline. Thirty to one hundred thousand events were analysed with a FACSCalibur^TM^ (Becton Dickinson) using the CellQuestPro^TM^ software analysis programme (Becton Dickinson). The CD1b^-^ L-DCs were analysed in a cell population gated on the basis of side scatter angle and RPE negative cells. Selected cells were then analysed for their expression of different markers.

CD1b^+^ and CD1b^-^ L-DCs were sorted using a fluorescence-activated cell sorter (MoFlo®, Beckman Coulter Inc., Brea, CA) as described previously [11]. After gating on a population negative for lymphocyte markers, positive and negative cells for CD1b were sorted. The proportion of the CD1b^+^ and CD1b^-^ L-DCs subsets were enriched from lymph, from 1% to 96%, and 15% to 99.7% respectively. 

### Stimulation of L-DCs with *Salmonella* and helminth secretions

The *Salmonella enterica* serovar Abortusovis Rv-6 strain, a live attenuated vaccine strain was used and prepared at the appropriate concentration to obtain an optimal multiplicity of infection (MOI) of 100 *Salmonella* per DC for each experiment. The excretory/secretory products of the nematode *Haemonchus contortus* (Hc-ES) were prepared from worms collected in sheep abomasum 21 days after experimental infestation. Briefly, worms were washed *in vitro* in DMEM (Life Technologies), incubated for 24h at 37°C and culture supernatant was filtrated on 0.2μm (Hc-ES kindly provided by C. Koch, Inra, Tours, France). The optimal concentration of Hc-ES used to stimulate L-DCs was defined between 150 and 200 μg of proteins/ml.

L-DCs were resuspended in complete medium without antibiotics, distributed in round-bottom plates (1 x 10^5^/well) and either infected with *Salmonella* or stimulated with Hc-ES. L-DCs were incubated for 60 min at 37°C with *Salmonella*, washed with antibiotics to remove and kill any remaining extracellular bacteria, and resuspended in complete medium supplemented with gentamicin (50 µg/ml) (100 µl), and incubated at 37°C for 6h or 18h as L-DCs stimulated with Hc-ES. L-DCs were washed and lysed with Lysis/Binding Buffer (Dynabeads, Oslo, Norway) (100 µl/1 x 10^5^ cells) and stored at -80°C.

### Antigen presentation assays using L-DCs and lymphocyte subset sorting

L-DCs were resuspended in complete medium without antibiotics, distributed in round-bottom plates (1 x 10^3^/well) and infected with *Salmonella* (MOI 100) or stimulated with Hc-ES (100 or 150 μg/ml) as described above, and incubated for 24h at 37°C. Autologous peripheral blood mononuclear cells (PBMC) were obtained from fresh blood as described previously [11] and added to the L-DCs (5 x 10^5^ cells/100 µl/well) and co-cultured for five days. This period was defined as optimal after a series of assays with different co-cultured times. CD4^+^ or CD8^+^ or γδ^+^ T cells were then positively sorted from cultured PBMC using immunomagnetic microbeads. PBMC were incubated either with anti- CD4 (17D1), anti- CD8 (CC58) or anti- γδ (86D) (2 µg/ml) mAb for 20 min, washed and incubated for a further 20 min with GAM IgG (H+ L)-coated magnetic microbeads in accordance with the manufacturer’s recommendations (GAM IgG MACS® microbeads, 130-048-402, Miltenyi Biotec, Paris, France). After three washes, cells were positively selected with an MS column and octoMACS™ separator (Miltenyi Biotec) and the efficiency of the resulting positive selection was checked using flow cytometry after incubating the sorted cells with RPE-conjugated GAM IgG. The purity of cell preparations was on average over 96% for CD4^+^ and CD8^+^ T cells, and 90% for γδ ^+^ T cells. Lymphocyte subsets were lysed with Lysis/Binding Buffer (Dynabeads) (100 µl/1 x 10^5^ cells) and stored at -80°C.

### RNA extractions and reverse transcriptase PCR

Messenger RNA was extracted from sorted CD1b^+^ or CD1b^-^ L-DCs and from CD4^+^ or CD8^+^ or γδ T cells using the Dynabeads® mRNA DIRECT™ Micro Kit (Invitrogen Dynal AS, Oslo, Norway). The mRNA was processed for reverse transcription and the generated cDNA was then analysed for the presence of sequences encoding for several molecules (Table 1) using real-time quantitative PCR (RT-qPCR) [11]. Samples were normalized internally using the cycle quantification (Cq) of GAPDH and HPRT simultaneously as references in each sample. Cq values were extracted with the RT-qPCR instrument software and subsequently imported into qbase^PLUS^ (http://www.qbaseplus.com) for quality control and generation of the standard curves. Relative quantities were calculated using the qBase quantification model which enables PCR efficiency correction, multiple reference assay normalization, proper error propagation and, if necessary, inter-run calibration [12].

**Table 1:** Primers used for PCR and real-time qPCR analysis.

**Target mRNA**	**Primer sequence**	**Annealing temperature (°C)**	**PCR product (bp)**	**Accession number**
Jagged-1	S: ACGTAGCTTGCGAGCCTTC	60	140	DQ152971
	AS : GTTCCCGTCACGTTTACTG			
Delta-4	S : GGGCAACATGCTCCAACAG	60	159	DQ152946
	AS : ACAGGCAGTGGTAGCCGTC			
GATA-3	S : CCCGTCCTACTACGGAAAC	60	193	BC123555
	AS : GTGGTGGATGGACGTCTTG			
T-Bet	S : TGTGGTCCAAGTTTAATCAGCA	60	287	DQ15299
	AS : TTTCCCGAATGACACCTCCT			
TNF-α	S : ACACTCAGGTCATCTTCTCAA	60	192	NM1024860
	AS : GAGGACCTGCGAGTAGATGAG			
IL1-ß	S : CAGGCAGGTAGTGTCGGTCAT	60	135	D63351
	AS : GATGTTTCGAAGATGACAGG			
IL-6	S : GGTGATGACTTCTGCTTTCC	60	204	NM001009392
	AS : TTTTCTGCCAGTGTCTCCTT			
IL-12p40	S : AACCTGCAACTGAGACCACT	62	186	NM001009438
	AS : ATCCTTGTGGCATGTGACTT			
IL-23	S : ATGGCTGTGATCCACAAGG	58	165	EU616677
	AS : CCAGTATGGAGGCGTGAAG			
IL-4	S : ATGTACCAGCCACTTCGTCC	60	120	XM004008636
	AS : ACGTCTGCTACAGGCAGCTC			
IL-13	S : CTGTGCAATGGCAGCATGG	60	151	DQ679798
	AS : TAGCTGAGGGCTTGTGAGG			
IL-17	S : TCCATCTCACAGCGAGCACAAG	60	113	Raffatallu*
	AS : AGCCACCAGACTCAGAAGCAGTAG			
IL-10	S : AGCAGCTGTACCCACTTCC	60	132	NM001009327
	AS : CAGCAGAGACTGGGTCAAC			
IFN-γ	S : TTCCGGTGGATGATCTGC	60	148	EF375708
	AS : GAGAACCATTACATTGATGCTC			
GAPDH	S: GGCGTGAACCACGAGAAGTATAA	60	119	NM001190390
	AS: CCCTCCACGATGCCAAAGT			
HPRT	S: AAACCAAAGATGGTCAAGGT	56	200	BC103248
	AS: TCTTAGGCTTTGTATTTTGCTT			

* Rafatallu M et al (2007) Infect. Immun. 75: s4342-4350

### Statistical analyses

In the experiments performed with L-DCs from several sheep, statistical analysis was carried out with the package “nparLD” (http://www.r-project.org/) designed to perform non-parametric analysis of longitudinal data in factorial experiments [13]. Non parametric variance analysis for repeated experimental designs was used to analyse the “CD1b phenotype” and “antigen” effects. 

## Results

### CD1b^+^ L-DC responds differently to *Salmonella* and helminth secretions

**Figure 1:**
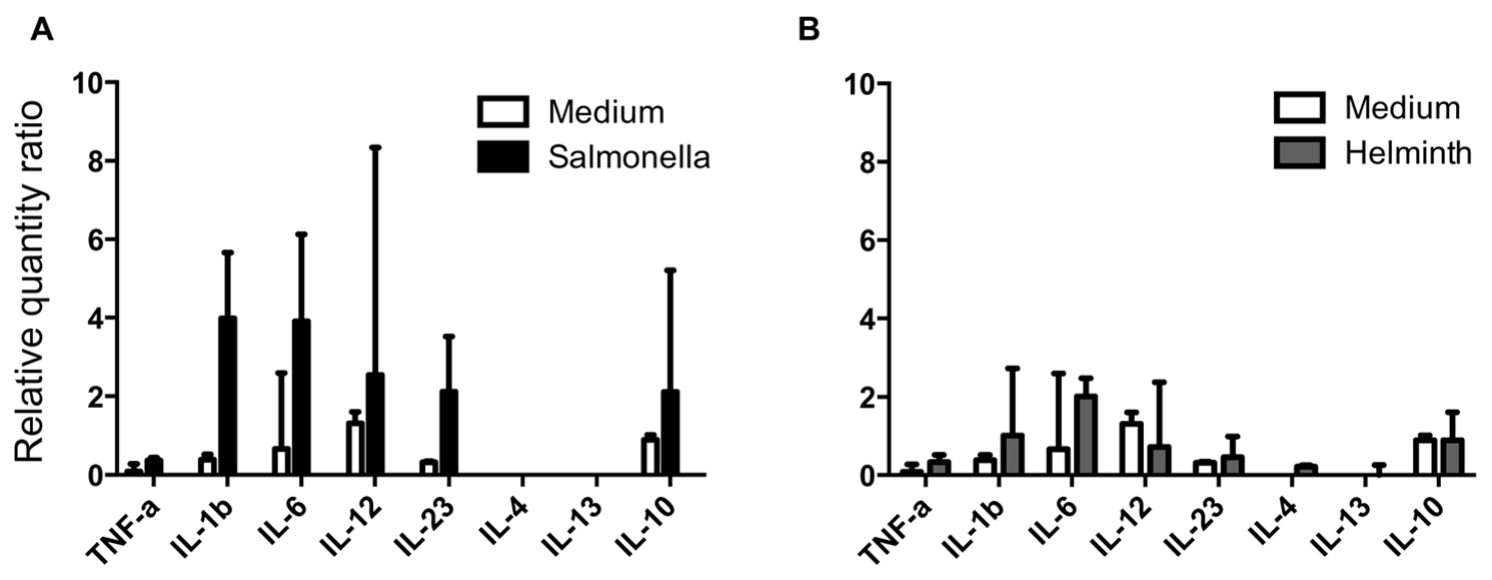
Cytokine response of CD1b^+^ L-DCs to Salmonella and helminth secretions. Production of cytokines was expressed as relative quantity ratios of RT-qPCR performed on mRNA extracted from cells incubated with *Salmonella* or Hc-ES for 6h. Data express the median (quartiles) from three sheep. (*) significantly different.

To characterize the flexible response of mature conventional CD1b^+^ L-DCs to pathogens, we analysed the cytokine mRNA produced by CD1b^+^ L-DCs after infection with *Salmonella* or stimulation with Hc-ES. CD1b^+^ L-DCs constitutively expressed IL-6, IL-12p40 and IL-10 mRNA, and to a lesser extent IL1-ß, TNF-α and IL-23 mRNA. The infection of CD1b^+^ L-DCs with *Salmonella* for 6h induced a significant increase in IL-1ß, IL-6, IL-12p40 and IL-10 mRNA, whereas only IL-6 mRNA increased after Hc-ES stimulation (Figure 1A, 1B). Only IL-12p40 mRNA was observed to increase significantly 18h after the infection of CD1b^+^ L-DCs with *Salmonella* (data not shown). In addition to the cytokine production by DCs, the activation of a cascade of transcription factors is important for the polarization of Th cells. To investigate the role of Notch ligands expressed by DCs in response to pathogens, and in possible polarized cytokine T cell response [14], we analysed the expression of the Notch ligands Jagged-1 and Delta-4 by CD1b^+^ L-DCs before and after infection with *Salmonella* or stimulation with Hc-ES. Only the production of Jagged-1 mRNA by CD1b^+^ L-DCs was detected, generally reported as promoting Th2 polarization [15].

These results show the capacities of CD1b^+^ L-DCs to promote different responses to two pathogens, characterized by a pro-inflammatory (IL-1ß, IL-6), pro-Th1 (IL-12p40) and IL-10 response to *Salmonella*, and a very limited pro-inflammatory (IL-6) response to helminth secretions.

### CD1b^-^ lymph cells are potential DCs which react differently than CD1b^+^ L-DC to *Salmonella* and helminth secretions

**Figure 2:**
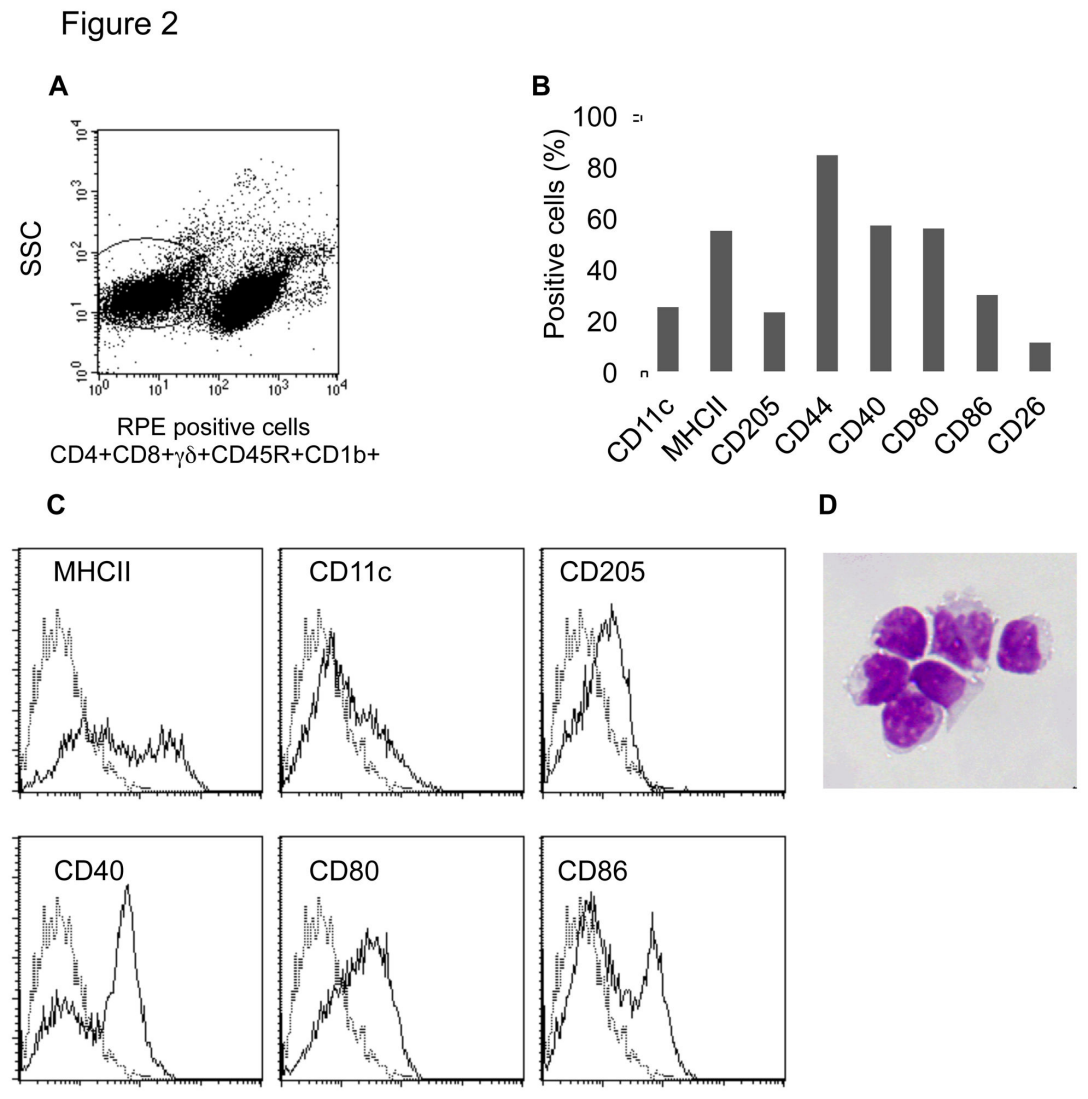
Phenotypic characterization of migratory CD1b^-^ L-DCs. After labelling of lymph cells with mAbs to CD4, CD8, γδ, CD45R and CD1b molecules, CD1b^-^ L-cells were gated on the negative labelled population and a low side scatter signal (A), and then analysed for the expression of different markers (B). Plots of different markers expressed by CD1b^-^ L-DCs are shown (C). May-Grünwald Giemsa staining of sorted and cytocentrifugated CD1b^-^ L-DCs (D).

Other than the CD1b^+^ L-DCs, the major population (90%) of cells not including T or B cells in the lymph did not express the CD1b molecule (Figure 2A, 2D). The phenotype of this cell population was characterized by analysing the expression of different markers. The majority of the population expressed MHCII, CD44, co-stimulatory molecules CD40 and CD80, and 20% expressed CD11c, CD205 and CD86 (Figure 2B, 2C). None of this cell population expressed CD14, CD11b, CD206, SIRP-α, and no CD103 or DC-SIGN mRNA was detected. Overall, CD1b^-^ lymph cells displayed phenotypic features of DCs, but were less mature than CD1b^+^ L-DCs. As these CD1b^-^ lymph cells are able to present *Salmonella* or helminth antigens to specific effector/memory T cells (unpublished data), this cell population was thereafter referred as CD1b^-^ L-DCs.

**Figure 3:**
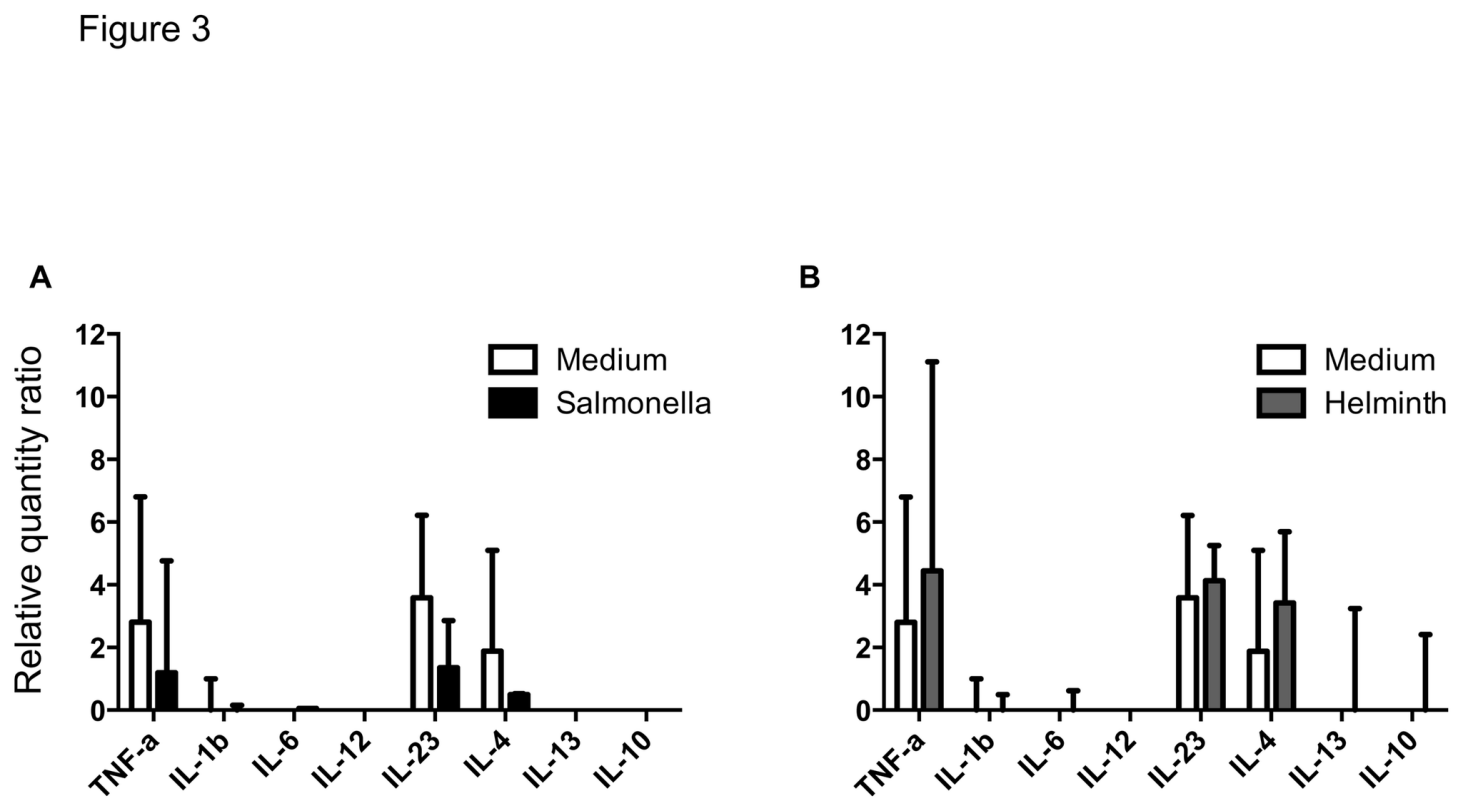
Cytokine response of CD1b^-^ L-DCs to *Salmonella* and helminth secretions. Production of cytokines was expressed as relative quantity ratios of RT-qPCR performed on mRNA extracted from cells incubated with *Salmonella* or Hc-ES for 6h. Data express the median (quartiles) from three sheep. (*) significantly different.

In contrast to CD1b^+^ L-DCs, CD1b^-^ L-DCs constitutively produced TNF-α, IL-23 and IL-4 mRNA (Figure 3 A). This constitutive production of TNF-α and IL-4 reduced significantly 6h after *Salmonella* infection, whereas it was maintained 6h after Hc-ES stimulation (Figure 3A, 3B), and then decreased 12h later (data not shown). The CD1b^-^ L-DCs did not express Notch ligands Jagged-1 and Delta-4 mRNA but constitutively expressed the transcription factor GATA-3 mRNA, whose production tended to fall after *Salmonella* infection (data not shown). 

Unlike CD1b^+^ L-DCs, CD1b^-^ L-DCs were constitutively predisposed to promote a Th17 and Th2 response which were down-regulated after *Salmonella* infection but not after stimulation with helminth secretions.

### Promotion of T cell subset cytokine responses by *Salmonella*-driven L-DCs

**Figure 4:**
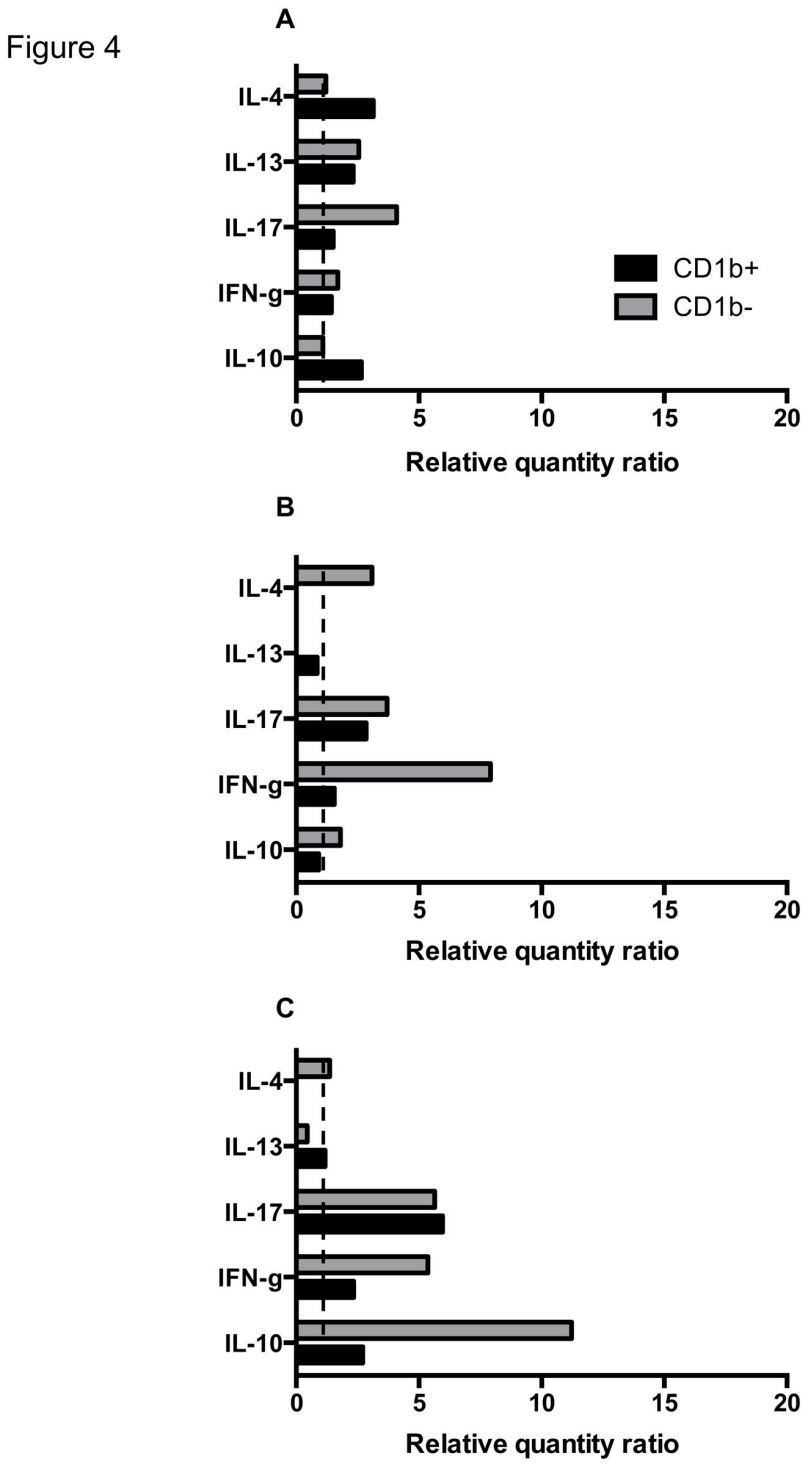
Promotion of T cell subset cytokine responses by *Salmonella*-driven L-DCs. Sorted CD1b^+^ and CD1b^-^ L-DCs were infected with *Salmonella* and co-cultured with PBMC at a ratio of 1:500 for 5 days. CD4^+^, CD8^+^ and γδ^+^ T cells were then positively sorted from cultured PBMC and mRNA extracted. Production of cytokines was expressed as relative quantity ratios of RT-qPCR performed on mRNA extracted from CD4^+^ (A), CD8^+^ (B), γδ^+^ T cells (C). Results from one representative experiment are shown.

The fate of T cells is determined by signals provided by DC subsets. To investigate the cytokine response of T cell subsets induced by L-DCs, we developed an *in vitro* model of L-DCs driven by *Salmonella*, co-cultured with PBMC to reproduce better the interaction of lymphocyte subsets which occurs *in vivo*. The expression of mRNA was then analysed after 5 days of co-culture on sorted CD4^+^, CD8^+^ and γδ^+^ T cells. One of these experiments is represented in the Figure 4. Cytokine mRNA expressed by T cell subsets in relation with the L-DC subsets tested was analysed qualitatively. The cytokine mRNA expressed the most by T cell subsets after co-culture with *Salmonella*-driven CD1b^+^ L-DCs was IL-17 (γδ ^+^ and CD8^+^ T cells), and to a lesser extent, IFN-γ (γδ^+^ T cells), IL-10 (CD4^+^ and γδ^+^ T cells), followed by IL-4 and IL-13 (CD4^+^ T cells). 

Although CD1b^-^ L-DCs were constitutively predisposed to promote the Th2 response, the main cytokine mRNAs produced by T cell subsets after co-culture with these *Salmonella*-driven DCs were IL-10 (γδ^+^ T cells), IFN-γ (CD8^+^, γδ^+^ T cells) and IL-17 (γδ^+^, CD4^+^, CD8^+^ T cells).

The expression of transcription factors T-Bet and GATA-3 are known to be involved in Th1 and Th2 polarization response of T cells respectively [16]. The mRNA expression of these molecules was relatively low in T cell subsets, with only GATA-3 mRNA detected in CD4^+^ and CD8^+^ T cells in response to *Salmonella*-driven CD1b^+^ L-DCs, and in CD4^+^ and γδ^+^ T cells in response to *Salmonella*-driven CD1b^-^ L-DCs (data not shown). 

These results support preferential IL-17, IFN-γ and IL-10 production by T cell subsets in response to *Salmonella*-driven CD1b^+^ L-DCs, and IL-10, IFN-γ and IL-17 production in response to *Salmonella*-driven CD1b^-^ L-DCs. 

### Promotion of T cell subset cytokine responses by helminth secretions-driven L-DCs

In addition to the role of L-DC subsets in the orientation of the T cell response, we examined the impact of pathogen – DC interaction in this orientation. For this, the same *in vitro* model of L-DCs – PBMC co-cultures was used to analyse the T cell subset response after stimulation with Hc-ES. 

**Figure 5:**
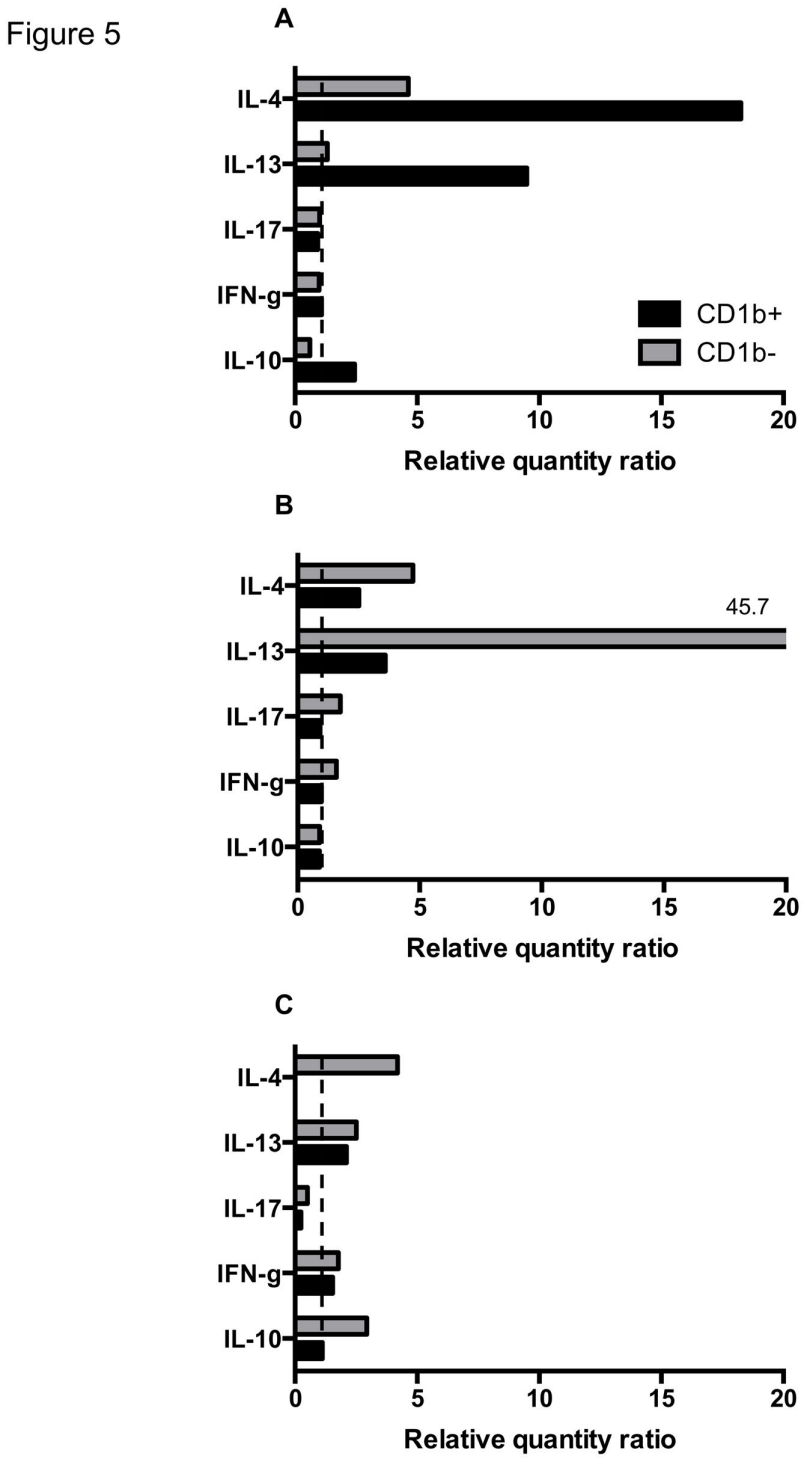
Promotion of T cell subset cytokine responses by helminth secretion-driven L-DCs. Sorted CD1b^+^ and CD1b^-^ L-DCs were stimulated with Hc-ES and co-cultured with PBMC at a ratio of 1:500 for 5 days. CD4^+^, CD8^+^ and γδ^+^ T cells were then positively sorted from cultured PBMC and mRNA extracted. Production of cytokines was expressed as relative quantity ratios of RT-qPCR performed on mRNA extracted from CD4^+^ (A), CD8^+^ (B), γδ^+^ T cells (C). Results from one representative experiment are shown.

The main cytokine mRNA expressed by T cell subsets after co-cultured with CD1b^+^ L-DCs driven by Hc-ES were IL-4 and IL-13 in CD4^+^ and to a lesser extent in CD8^+^ T cells (Figure 5). IL-13 mRNA was predominantly expressed in CD8^+^ T cells in response to Hc-ES-driven CD1b^-^ L-DCs, whereas IL-4 mRNA was expressed in both CD4^+^, CD8^+^ and γδ^+^ T cell subsets. The expression of GATA-3 mRNA was detected in all T cell subsets in response to Hc-ES-driven L-DCs (data not shown).

These results are support the predominant promotion of the Th2 response by Hc-ES-driven L-DCs, both for CD1b^+^ and CD1b^-^ L-DCs.

## Discussion

The present study investigated *in vitro* the flexible capacity of physiologically generated L-DCs to respond to different pathogens, *Salmonella* and helminth secretions, and their impact on the promotion of the cytokine response in T cell subsets. Mature conventional CD1b^+^ L-DCs showed a predisposition to promote pro-inflammatory (IL-6), pro-Th1 (IL-12p40) and anti-inflammatory (IL-10) responses which were amplified in response to *Salmonella* and associated with a preferential production of pro-inflammatory IL-17, and to a lesser extent IFN-γ and IL-10 by T cells. In contrast to *Salmonella*, CD1b^+^ L-DCs driven by Hc-ES induced a very limited gene expression associated with a Th2 (IL-4 and IL-13) response by T cells. The characterization of the CD1b^-^ L-DCs showed a predisposition of these immature DCs to promote a Th2 (IL-4) or Th17 (IL-23) response, which was associated with a Th2 (IL-4 and IL-13) response by T cells when CD1b^-^ L-DCs were stimulated with Hc-ES. These CD1b^-^ L-CDs were also able to modify their response to *Salmonella* and to promote IL-10, IFN-γ and IL-17 expression by T cell subsets.

The *Salmonella*-driven CD1b^+^ L-DCs were able to promote preferentially the gene expression of IL-17, IFN-γ and IL-10 in T cells. IL-17 gene expression was mainly observed in γδ^+^ and CD8^+^ T cells but not in CD4^+^ T cells. These results are partly in line with the production of IL-17 observed after systemic infection of mice with attenuated *Salmonella enterica* serovar Enteritidis [7]. In that study, IL-17 was produced by Th17, γδ^+^ T cells and other CD4^-^ lymphocytes. In our study, CD4^+^ T cells did not express IL-17 gene whereas γδ^+^ T cells and CD8^+^ T cells did. Production of IL-17 by CD4^-^ T cells has also been reported *in vivo* during the early stages after *Mycobacterium tuberculosis* infection suggesting this response occurs before αß^+^ T cell priming or substantive IFN-γ production [17,18]. Our results suggest that this is what occurred in the present *in vitro* study as IL-23 transcripts in CD1b^+^ L-DCs were detected after *Salmonella* infection, but possibly in insufficient quantities to promote IL-17 production by CD4^+^ T cells. IFN-γ gene expression was also observed only in γδ^+^ T cells and not CD4^+^ T cells. This could be related to the system of PBMCs co-culture chosen to be physiologically closer to the *in vivo* situation, and which highlighted the T cell subsets predominantly promoted by *Salmonella*-driven L-DCs. In a previous study, we showed that *Salmonella*-driven CD1b^+^ L-DCs were able to promote IFN-γ production by CD4^+^ T cells [11] which is known to be essential for controlling *Salmonella* growth [5]. As IL-17 promotes neutrophils influx into infected or damaged tissues, this early production of IL-17 by γδ^+^ T cells would contribute to the first line of defence against *Salmonella in vivo* and complement the role of neutrophils in the lymphatic transport of *Salmonella* from tissues to lymph nodes [19]. This predominant pro-inflammatory T cell response promoted by *Salmonella*-driven CD1b^+^ L-DCs has also been shown to be associated with anti-inflammatory IL-10 gene expression in γδ^+^ and CD4^+^ T cells, cytokines playing an important role in generating regulatory environments [20].

The flexible response of DCs to pathogens has mainly been reported for monocyte-derived DCs revealing the capacity of these cells to sense diverse pathogens, and to elicit common and differential pathogen recognition pathways [21]. To evaluate the flexible capacity of migrating physiologically generated conventional mature DCs, we chose to test the reactivity of CD1b^+^ L-DCs to helminth antigens known to induce a Th2 response, and for which few specific data are available regarding interactions of DC subsets with helminths. Compared to the response of *Salmonella*-driven CD1b^+^ L-DCs, their response to helminth secretions was much attenuated and limited to an increase in IL-6 gene expression. This is in line with the overall down-regulation of immune gene expression observed in lymph in response to gastrointestinal nematodes [22]. These Hc-ES-driven CD1b^+^ L-DCs were able to promote preferentially the transcript expression of the Th2 cytokines, IL-4 and IL-13, in CD4^+^ T cells and to a lesser extent in CD8^+^ T cells, linked to the expression of the transcription factor GATA-3 gene. These results confirm that the Th2 response to helminth antigens does not occur by default, due to the lack of conventional characteristics of DC maturation [20,23,24], but can occur in the presence of the DC phenotype expressing the transcription factor Jagged-1 gene, and of DCs able to drive a pro-inflammatory IL-17 and IFN-γ response to *Salmonella*.

Ruminant L-DCs were originally defined on the expression of the CD1b and CD14 molecules [25] comprising CD8α-like DCs [26]. CD1b belongs to the group 1 of CD1 isoforms involved more in presenting foreign lipid antigens, and traffic from the cell surface through late endosomes and lysosomes, colocalizing in specialized lysosomes with MHCII [27]. However, the major population of cells, not including T or B cells, in lymph did not express the CD1b molecule. We characterized the phenotype of these cells expressing, for the majority, MHCII, CD44 and co-stimulatory CD40 and CD80 molecules which play an important role in dC-T interactions and subsequent T cell activation [28], and for 20% of them CD11c, CD205 and CD86. They did not express CD14, CD11b or CD206 molecules. This heterogeneous cell population includes plasmacytoid DCs (CD45RB+ CD1b^-^ CD11c^-^ CD11b^-^)[29] and possibly a number of tissue-derived DC subsets. The presence of innate immune cells [30] in these CD1b^-^ lymph cells cannot be excluded, but, to our knowledge, no data are available on the characteristics of these cells in the lymph. The CD1b^-^ L-DCs constitutively expressed GATA-3, TNF-α, IL-23 and IL-4 genes which were down-regulated after *Salmonella* infection, but not after stimulation with helminth secretions. These Hc-ES-driven DCs were able to promote preferentially the expression of IL-13 gene in CD8^+^ T cells and of IL-4 in all T cell subsets. Surprisingly, *Salmonella*-driven CD1b^-^ L-DCs were able to promote, in order of priority, the transcript expression of IL-10 in γδ^+^ T cells, IFN-γ in both CD8^+^ and γδ^+^ T cells, and IL-17 in all T cells. The gene expression in T cells by *Salmonella*-driven CD1b^-^ L-DCs tended to be higher than that promoted by CD1b^+^ L-DCs. These results show that although predisposed to promoting the Th2 response, the CD1b^-^ L-DCs were capable of promoting anti-inflammatory (IL-10) and pro-inflammatory (IFN-γ and IL-17) responses to *Salmonella*. To analyse further the influence of the environment on the orientation of T cell responses by L-DCs, we used mucosal L-DCs collected from cervical lymph after cannulation to compare their response to those of cutaneous L-DCs. The CD1b^+^ and CD1b^-^ L-DCs of mucosal origin did not modify the promotion of the T cell subset cytokine response driven by *Salmonella* or helminth secretions compared to cutaneous L-DCs (unpublished data).

Overall, *Salmonella*-driven L-DCs preferentially promoted a pro-inflammatory T cell response (IFN-γ and IL-17) via the CD1b^+^ L-DCs and an anti-inflammatory (IL-10) and IFN-γ T cell response via the CD1b^-^ L-DCs. Helminth secretion-driven L-DCs preferentially promoted a Th2 response, IL-4 via CD1b^+^ L-DCs and IL-13 via CD1b^-^ L-DCs. Overall, the promotion of the T cell response was controlled more by the type of pathogen which interacted with the L-DCs than the L-DC subsets. The main conclusion of this study is that the migrating L-DC subsets, whatever their degree of maturity or programming, are able, at least *in vitro*, to reprogram different gene expression profiles according to the pathogen encountered, and to promote tailored pathogen-specific immune responses. These flexible capacities of migrating DCs could be used in vaccine strategies for driving accurate immune responses.
